# Can a national government implement a violence prevention and response strategy for key populations in a criminalized setting? A case study from Kenya

**DOI:** 10.1002/jia2.25122

**Published:** 2018-07-22

**Authors:** Parinita Bhattacharjee, Giuliana J Morales, Timothy M Kilonzo, Robyn L Dayton, Reuben T Musundi, Janet M Mbole, Serah J Malaba, Bernard E Ogwang, Shajy K Isac, Stephen Moses, Helgar K Musyoki

**Affiliations:** ^1^ Centre for Global Public Health University of Manitoba Winnipeg Canada; ^2^ FHI 360 Durham USA; ^3^ Partners for Health and Development in Africa Nairobi Kenya; ^4^ National AIDS Control Council Ministry of Health Nairobi Kenya; ^5^ FHI 360 Nairobi Kenya; ^6^ National AIDS and STI Control Programme Ministry of Health Nairobi Kenya

**Keywords:** key populations, violence response, Government of Kenya, HIV, enabling environment

## Abstract

**Introduction:**

Key population (KP) members frequently experience violence that violates their human rights, increases their risk of HIV, and acts as a barrier to access and uptake of HIV services. To be effective, HIV programmes for members of KPs need to prevent and respond to violence against them. We describe a violence prevention and response strategy led by the national KP programme in Kenya and examine trends in reports of and responses to violence (provision of support to an individual who reports violence within 24 hours of receiving the report).

**Methods:**

Quarterly programme monitoring data on the number of reports of violence and the number of responses to violence from 81 implementing partners between October 2013 and September 2017 were aggregated annually and analysed using simple trend analysis. Reports of violence relative to KP members reached, expressed as a percentage, and the percentage of reports of violence that received a response were also examined.

**Results and Discussion:**

Between 2013 and 2017, annual reports of violence increased from 4171 to 13,496 cases among female sex workers (FSWs), 910 to 1122 cases among men who have sex with men (MSM) and 121 to 873 cases among people who inject drugs (PWID). Reports of violence relative to KP members reached increased among FSWs (6.2% to 9.7%; *p* < 0.001) and PWID (2.1% to 6.0%; *p* < 0.001) and decreased among MSM (10.0% to 4.2%; *p* < 0.001). During the same period, timely responses to reports of violence increased from 53% to 84% (*p* < 0.001) among FSWs, 44% to 80% (*p* < 0.001) among MSM and 37% to 97% (*p* < 0.001) among PWID.

**Conclusions:**

Over the past four years in Kenya, there has been an increase in violence reporting among FSWs and PWID and an increase in violence response among all KPs. This case study demonstrates that violence against KP members can be effectively addressed under the leadership of the national government, even in an environment where KP members’ behaviours are criminalized. Creating an enabling environment to promote wellbeing and safety for KP members is a critical enabler for HIV prevention programmes to achieve 95‐95‐95 goals.

## 1 Introduction

The HIV Prevention 2020 Roadmap provides guidance and demonstrates commitment from African countries to accelerate interventions to reduce new HIV infections by 75% 1. In Africa, the proportion of new infections among key populations (KPs), defined as female sex workers (FSWs), men who have sex with men (MSM), and people who inject drugs (PWID), is substantial 2. Greater investment in KP programmes can improve the effectiveness of HIV interventions 3. However, punitive laws related to sex work, same‐sex sexual practices and drug use; stigma and discrimination in community and healthcare settings; and violence hinder access to and uptake of HIV‐related services among KPs 4, 5, 6. In such environments, UNAIDS has called upon governments to create an enabling environment and develop pragmatic solutions to ensure that KPs can organize to reduce risk and prevent HIV, while simultaneously increasing access to prevention programmes 1.

Kenya has a mixed and geographically heterogeneous HIV epidemic, with an estimated national adult HIV prevalence of 5.9% 7, and an estimated 33% of all new infections occurring among KP members 8. In Nairobi, HIV prevalence is 6.1% among adults, 29.3% among FSWs, 18.2% among MSM and 18.7% among PWID 7, 9. The KP programme is led by the National AIDS and STI Control Programme (NASCOP) and the National AIDS Control Council (NACC) within the Ministry of Health and implemented by 81 partners primarily funded by PEPFAR and the Global Fund. NACC and NASCOP, in collaboration with KP‐led organizations, have developed policies 10 and guidelines 11 to support KP programme implementation. The programme has been scaled up to reach an average of 139,041 FSWs in 33 counties, 26,972 MSM in 29 counties and 14,527 PWID in 16 counties quarterly 12. A national population‐based survey conducted by NASCOP with 5,353 KPs in 2017 found that, in the last three months, 88% of FSWs, 80% of MSM and 84% of PWID were met by a peer educator; 85% of FSWs, 76% of MSM and 74% of PWID received an HIV test; and 74% of FSWs, 68% of MSM and 72% of PWID accessed services from a programme site 13.

Research in Kenya has found that physical, sexual and emotional violence against KPs is common and frequently perpetrated by sex work clients, police, religious leaders, intimate partners and strangers 14, 15, 16, 17, 18, 19, 20, 21, 22, 23, 24. A survey conducted by NASCOP in 2017 found that 22% of FSWs, 14% of MSM and 12% of PWID experienced physical or sexual violence, while 48% of FSWs, 20% of MSM and 44% of PWID experienced police violence in the past six months 13. Each population has unique experiences with violence: violence against FSWs is often physical violence perpetrated by clients and regular sex partners, while violence against MSM is often “opportunistic aggression” perpetrated by strangers 15. Furthermore, MSM who engage in sex work are two times more at risk of violence than those who do not 25. Violence against PWID is often associated with cocaine or heroin use and includes arbitrary police sweeps, beatings, harassment, bribery, remand, and imprisonment 20. Across populations, violence is fuelled by gender inequalities and stigma and discrimination against persons perceived to depart from conventional gender and sexual norms and identities 26, 27, 28. The Kenyan legal system and county by‐laws that criminalize behaviours related to sex work, same‐sex sexual practices and drug use also legitimize violence, stigma and discrimination against KPs 29, 30, 31, 32.

Violence against KP members increases HIV risk and decreases HIV service uptake 33, 34, 35, 36, 37. Studies in Kenya show that violence and HIV are linked through intermediate risk factors such as unprotected sex and unsafe injecting practices 20, 38, 39. One study found that female and male sex workers who anticipate sexual violence or experience physical violence are more likely to avoid or delay HIV services than those who do not 24. Experiencing violence or fear of violence can also result in KPs prioritizing their safety over less immediate concerns such as HIV 40, 41. Furthermore, modelling in Kenya suggests that eliminating sexual violence against FSWs could avert 17% of new HIV infections among FSWs and their clients over 10 years 38.

The Kenyan national KP programme has prioritized violence as a key structural barrier to HIV‐related services 11, and adopted strategies to address violence against KPs in the National Guidelines for HIV/STI Programming with KPs 11. The KP programme posits that increasing violence reporting by KP members and improving responses to violence by implementing partners will reduce violence and thereby reduce new HIV infections among KP members in the long run 3, 11, 42. In this paper, we describe a violence prevention and response strategy adopted by the KP programme and examine trends in reports of and responses to violence.

## 2 Methods

### 2.1 Data Source

Eighty‐one implementing partners who serve FSWs, MSM and PWID, and operate in primarily urban areas, collected individual‐level service utilization data monthly (number of KPs reached, number of reports of violence and number of responses to violence) and submitted quarterly, aggregate‐level information to NASCOP as part of routine programme monitoring between October 2013 and September 2017. A report of violence is defined as a KP member disclosing violence — such as sexual assault, physical assault, verbal abuse, discrimination and arbitrary arrest — to a violence response team member via a helpline, a peer educator during outreach or a clinician during a facility visit. A response to violence is defined as a violence response team member, peer educator or clinician providing first‐line support within 24 hours of receiving the report to the individual who disclosed violence. First‐line support includes active listening, providing key messages and information on rights, safety planning, and providing services directly or through referrals.

### 2.2 Analysis

Number of KPs reached, number of reports of violence and number of responses to violence were aggregated annually and analysed. The rates of increase in KP members reached and reports of violence were analysed using the reported numbers in 2013 to 2014 as the reference year, and then dividing the numbers from subsequent reporting years over the reference year. The 2013 to 2014 period was selected as the reference year as it was before the violence prevention and response strategy was introduced in 2014 to 2015. Reports of violence relative to KP members reached, expressed as a percentage, and the percentage of reports of violence that received a response were also calculated. A chi square test was used to determine whether changes in violence reporting and response over time were statistically significant.

### 2.3 Ethics approval

These data were collected through routine programme monitoring and cannot be linked to any individual. Ethical approval to conduct secondary data analysis was received from the Kenyatta National Hospital — University of Nairobi Ethical Review Committee, number P647/11/2017.

### 2.4 Violence prevention and response strategy

**Figure 1:**
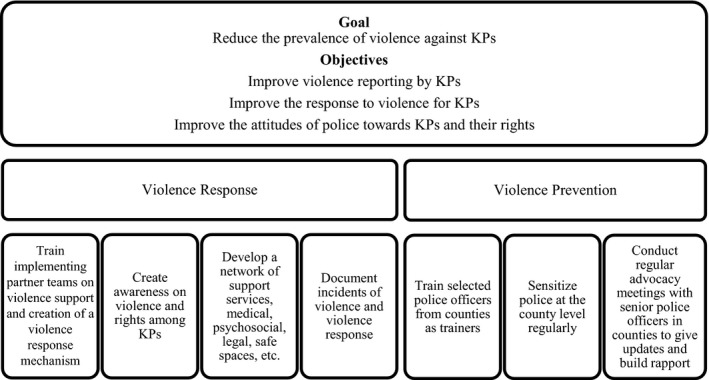
Two‐pronged strategy to address violence against key populations (KPs) in Kenya.

The national KP programme developed a violence prevention and response strategy (Figure 1) and complementary protocol 43, in consultation with KP‐led organizations, to guide NASCOP, NACC and implementing partners to develop and implement a violence prevention and response plan. Although the national KP programme has a two‐pronged strategy that includes violence prevention, this paper focuses on violence response.

Building implementing partner knowledge and skills to support KP victims of violence is central to violence response. Implementing partners were trained using a national curriculum developed by NASCOP 44, and in turn, educated KPs on violence and their rights using NASCOP‐supported communication materials during outreach and clinical services. Implementing partners conducted educational events at drop‐in centres to encourage reporting and service seeking and to set up mechanisms to effectively and efficiently respond to violence. Some implementing partners have violence response teams that manage 24‐hour helplines. The violence response teams consist of peer educators, peer paralegals and outreach workers that are supported by clinicians, advocacy officials and programme managers. Each implementing partner developed a network of services to ensure that post‐violence support (clinical, psychosocial, legal and safety) could be provided directly or through referral. KP members are encouraged to report violence by calling violence response team helplines, disclosing to a peer educator during outreach or disclosing to a clinician during a facility visit. When violence is disclosed, the violence response team member, peer educator or clinician seeks information about the incident and provides first‐line support. They assess the situation and, in collaboration with the victim, develop a response and support plan that includes further post‐violence support, per need and client preferences. They also document the incident of violence in a standard national format. In Kenya, the goal is to initiate post‐violence support within 24 hours of receiving a report.

## 3 Results and Discussion

Between 2013 and 2014 (reference year) and 2016 to 2017, the annual number of KP members reached increased from 67,432 to 139,041 among FSWs, 9118 to 26,972 among MSM and 5856 to 14,527 among PWID (Tables 1, 2, 3). During the same period, the annual number of reports of violence increased from 4171 to 13,496 among FSWs, 910 to 1122 among MSM and 121 to 873 among PWID. Reports of violence relative to KP members reached increased among FSWs (6.2% to 9.7%; *p* < 0.001) and PWID (2.1% to 6.0%; *p* < 0.001) and decreased among MSM (10.0% to 4.2%; *p* < 0.001). The percentage of reports of violence that received a response increased among all KPs: 53% to 84% among FSWs (*p* < 0.001), 37% to 97% among PWID (*p* < 0.001) and 44% to 80% among MSM (*p* < 0.001).

**Table 1:** Annual data on programme reach, reports of violence and responses to violence by FSWs in Kenya, October 2013 through September 2017

Indicator	Oct 2013 to Sept 2014^a^	Oct 2014 to Sept 2015	Oct 2015 to Sept 2016	Oct 2016 to Sept 2017
Number of FSWs reached	67432	88327	113096	139041
Number of FSW reports of violence	4171	5341	11707	13496
Rate of increase in number of FSWs reached	1.0	1.3	1.7	2.1
Rate of increase in number of FSW reports of violence	1.0	1.3	2.8	3.2
FSW reports of violence relative to FSWs reached, expressed as a percentage	6.2	6.0	10.4	9.7^b^
Percent of responses to FSW reports of violence	53	65	74	84^b^

a Reference year

b *p* < 0.001

**Table 2:** Annual data on programme reach, reports of violence and responses to violence by MSM in Kenya, October 2013 through September 2017

Indicator	Oct 2013 to Sept 2014^a^	Oct 2014 to Sept 2015	Oct 2015 to Sept 2016	Oct 2016 to Sept 2017
Number of MSM reached	9118	12106	16680	26972
Number of MSM reports of violence	910	849	1026	1122
Rate of increase in number of MSM reached	1.0	1.3	1.8	3.0
Rate of increase in number of MSM reports of violence	1.0	0.9	1.1	1.2
MSM reports of violence relative to MSM reached, expressed as a percentage	10.0	7.0	6.2	4.2^b^
Percent of responses to MSM reports of violence	44	77	86	80^b^

a Reference year

b *p* < 0.001

**Table 3:** Annual data on programme reach, reports of violence and responses to violence by PWID in Kenya, October 2013 through September 2017

Indicator	Oct 2013 to Sept 2014^a^	Oct 2014 to Sept 2015	Oct 2015 to Sept 2016	Oct 2016 to Sept 2017
Number of PWID reached	5856	7001	10990	14527
Number of PWID reports of violence	121	533	839	873
Rate of increase in number of PWID reached	1.0	1.2	1.9	2.5
Rate of increase in number of PWID reports of violence	1.0	4.4	6.9	7.2
PWID reports of violence relative to PWID reached, expressed as a percentage	2.1	7.6	7.6	6.0 b
Percent of responses to PWID reports of violence	37	48	89	97^b^

a Reference year

b *p* < 0.001

Violence response is relatively new in KP programmes, and it is vital to monitor whether such activities result in expected outcomes 45. In the absence of validated and internationally agreed‐upon indicators specific to violence against KPs, projects such as the *Avahan* AIDS Initiative in India collected data on reports of and responses to violence to monitor programme effectiveness 5, 6. Programmatic data on reports of violence are important because violence cannot be redressed if unreported 46, and reporting is a critical first step to link victims to post‐violence support 5, 6. Reports of violence over time also provide useful information about programme outcomes as increased reporting often occurs when KPs understand that violence was committed, know their rights, have an enabling environment to disclose violence and seek support, and have confidence in the response mechanism 5, 6, 46, 47. Programmatic data on responses to violence are also important as they show whether KPs who reported violence received critical post‐violence support such as post‐exposure prophylaxis 48. These data can also inform processes to address violence within the programme and guide the development of policies relevant to KPs 49.

Among FSWs and PWID, the increase in reports of violence relative to individuals reached may be due to the introduction of the national violence prevention and response strategy in 2014 to 2015. This is in line with global literature that shows that programmes tend to see an uptick in reports of violence after a programme monitoring system is established 5 and victims are sensitized 46. The increase in responses to reports of violence may also help explain this rise as FSWs and PWID may be more likely to report violence if they have social proof that their peers have received a positive response upon disclosing violence 50.

Among MSM, the decrease in reports of violence relative to individuals reached tells a different story. MSM‐serving organisations in Kenya established a system to ensure safety and security among MSM, sensitized police and religious leaders and conducted community outreach beginning in 2010 in reaction to violence against MSM in Mtwapa 23. This system was strengthened in anticipation of a backlash when Uganda's Anti‐Homosexuality Act was passed in 2014 23. This longer‐term effort, coupled with the high percentage of reports of violence in the reference year and decreases in reports in subsequent years, suggest that an uptick in reporting occurred before the strategy was introduced and that the decrease in reports of violence may reflect actual decreases in violence.

These programmatic data alone, however, have limited ability to show trends in actual violence. Fortunately, in Kenya, there is supplemental information on prevalence of physical/sexual violence and police violence against KP members from national population‐based surveys conducted 2014 to 2017 13. According to the surveys, prevalence of physical/sexual violence stayed static among FSWs, increased among PWID and decreased among MSM while police violence increased among FSWs and decreased among PWID and MSM 13. Programmatic and population‐based data offer useful information on their own and in relation to one another. For example, even at peak reporting, programmatic data on reports of violence have not reached the level of violence found in the surveys. This suggests that KPs may still be underreporting violence to implementing partners, for example, because there are remaining barriers to reporting, such as perceived stigma against victims 51, or because KPs are more equipped to address violence through their own networks without implementing partners 52.

### 3.1 Limitations

These programmatic data do not provide a full account of violence experienced by KPs in Kenya. For example, as the data were reported in aggregate on a quarterly basis, we were not able to conduct advanced statistical analysis, including exploring whether violence outcomes are related to HIV outcomes. As mentioned previously, programmatic data are limited in tracking actual violence and could overestimate violence (if some KPs report multiple incidents of violence) or underestimate violence (if KPs do not report violence they experience).

Going forward, it will be important to continue to collect and triangulate programmatic and population‐based data to analyse trends, including types of violence and perpetrators, over time. To address challenges in interpreting violence data, there is also a need for refined metrics and research to explore whether violence prevention and response interventions in KP programmes are having the intended effects, including specific questions on KP members’ experiences and satisfaction with violence prevention and response services. Future research also needs to explore whether an increase in responses to violence corresponds with an increase in HIV service uptake and whether those who report violence are more likely to re‐test or adhere to antiretroviral drugs than those who do not report violence.

## 4 Conclusions

The programmatic data from the national KP programme show that reports of violence relative to individuals reached increased among FSWs and PWID but decreased among MSM while the percent of reports of violence that received a response increased among all KPs. These data suggest that FSWs and PWID have the desire and ability to report violence, that MSM are seeing decreases in violence and that implementing partners are responding to reports of violence in a timely manner. This paints an overall positive picture of the impacts of the national violence prevention and response strategy and emphasizes the need for multiple sources of information to triangulate data and observe changes that may not become immediately apparent in programmatic or population‐based data alone.

In most parts of Africa, where sex work, same‐sex sexual practices and drug use are criminalized, implementing HIV programmes for KPs can be a challenge. However, Kenya has shown that with government leadership, it is not only possible to implement a robust key population programme, but also to address violence to help create an enabling environment for KPs to access HIV‐related services and thus enable the country to move more effectively towards meeting the 95‐95‐95 global targets.

## Funding

This publication is made possible by the support of BMGF under grant ID OPP 1032367 as well as the American People through USAID under cooperative agreement number AID‐OAA‐A‐14‐00045. The views expressed herein are those of the authors and do not necessarily reflect the official policy or position of BMGF, USAID, or the U.S. government.

## Authors’ contributions

PB and GJM were involved equally in the development of this manuscript. HKM, PB, TMK, JMM, RTM and SJM designed and implemented the intervention. PB and SKI were involved in analysing the quantitative data. RLD, SM, HKM and BEO were involved in reviewing and finalizing the manuscript. All authors have reviewed and approved the final manuscript.

## Competing interests

The authors declare that they have no competing interests.
